# Supplementary material for: Global variability in atmospheric new particle formation mechanisms
Source: Nature. 2024 Jun 12;631(8019):98–105. doi: 10.1038/s41586-024-07547-1 (PMC11222162; doi:10.1038/s41586-024-07547-1)
Supplement: Supplementary file 1 — This file contains Supplementary Figs. 1–12, Supplementary Tables 1–3 and further references. [file 41586_2024_7547_MOESM1_ESM.pdf]

nature portfolio

https://doi.org/10.1038/s41586-024-07547-1

Supplementary information

Global variability in atmospheric new particle formation mechanisms

In the format provided by the authors and unedited

Supplementary Information for

“Global variability in atmospheric new particle formation mechanisms”

This PDF file includes:

Supplementary Figs 1–12
Supplementary Table 1–3
Supplementary references

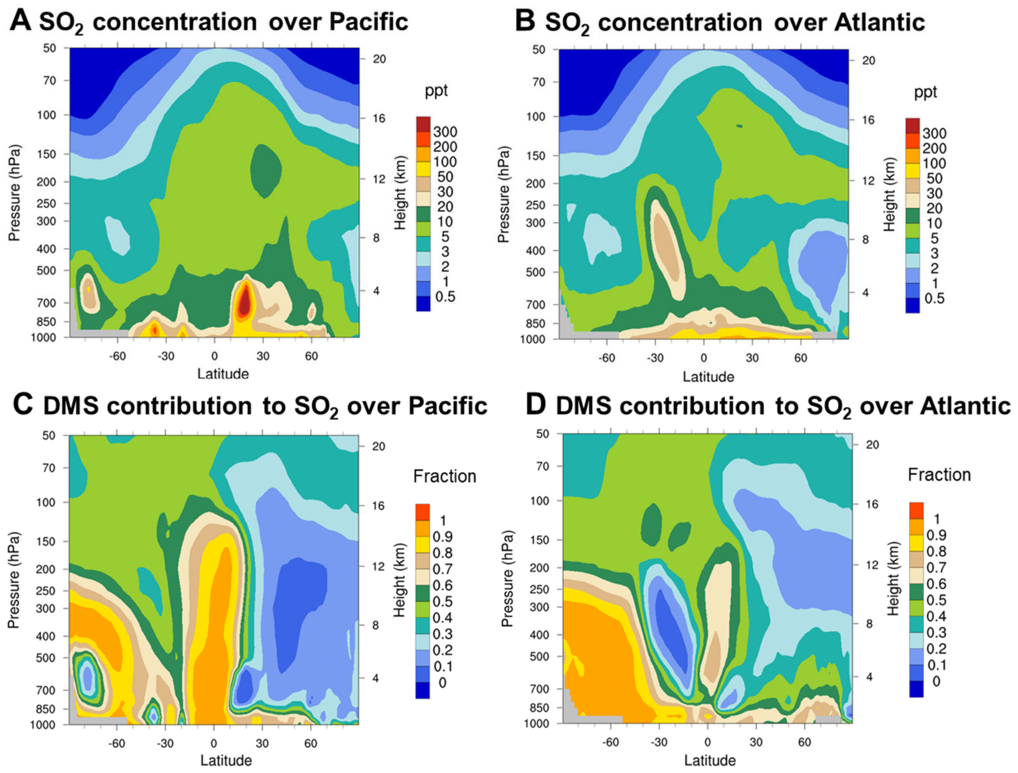

Supplementary Fig. 1 Zonal mean SO_2 concentrations and contribution of DMS emissions over the Pacific and Atlantic oceans in 2016. The range used for the Pacific Ocean is 170°E–150°W and the range used for the Atlantic Ocean is 20°–40°W. Contribution of DMS emissions is quantified using the difference between a simulation with zero DMS emission and the best-case simulation. In the tropical upper troposphere, oceanic DMS emissions account for >80% of the SO_2 and hence H_2SO_4 concentration over the Pacific and approximately 50% over the Atlantic, while continental anthropogenic SO_2 emissions control H_2SO_4 formation in the mid-latitude upper troposphere.

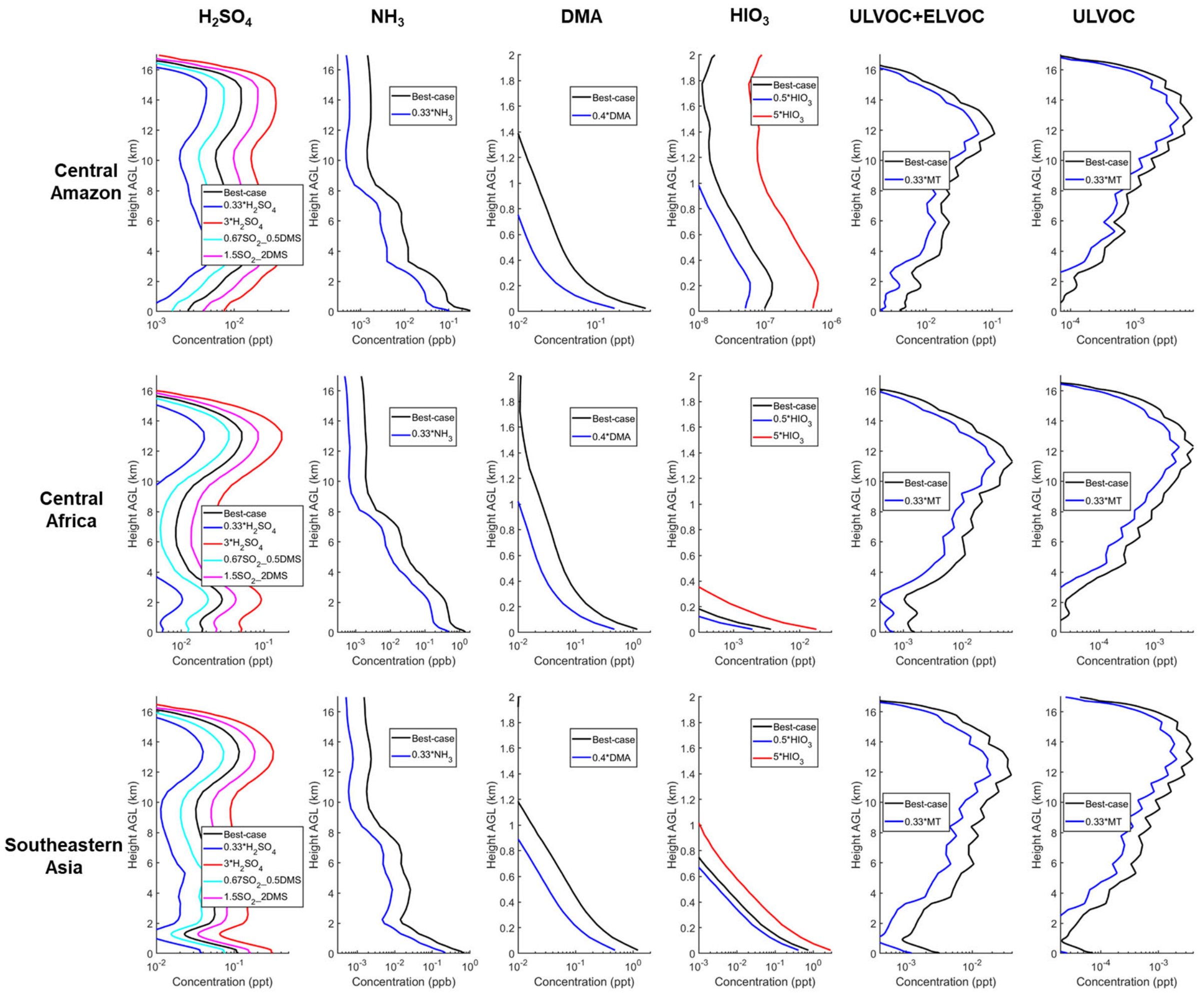

Supplementary Fig. 2 Concentrations of precursors directly involved in nucleation as a function of height AGL over rainforest regions under the best-case and sensitivity scenarios. The concentrations are averaged in 2016 over the regions specified in Extended Data Fig. 1B. Definitions of the sensitivity experiments are presented in Methods and Supplementary Table 1. To make the figures concise, we show only the concentrations of a precursor in the best-case simulation and in the sensitivity simulations where its concentrations show significant differences from the best case.

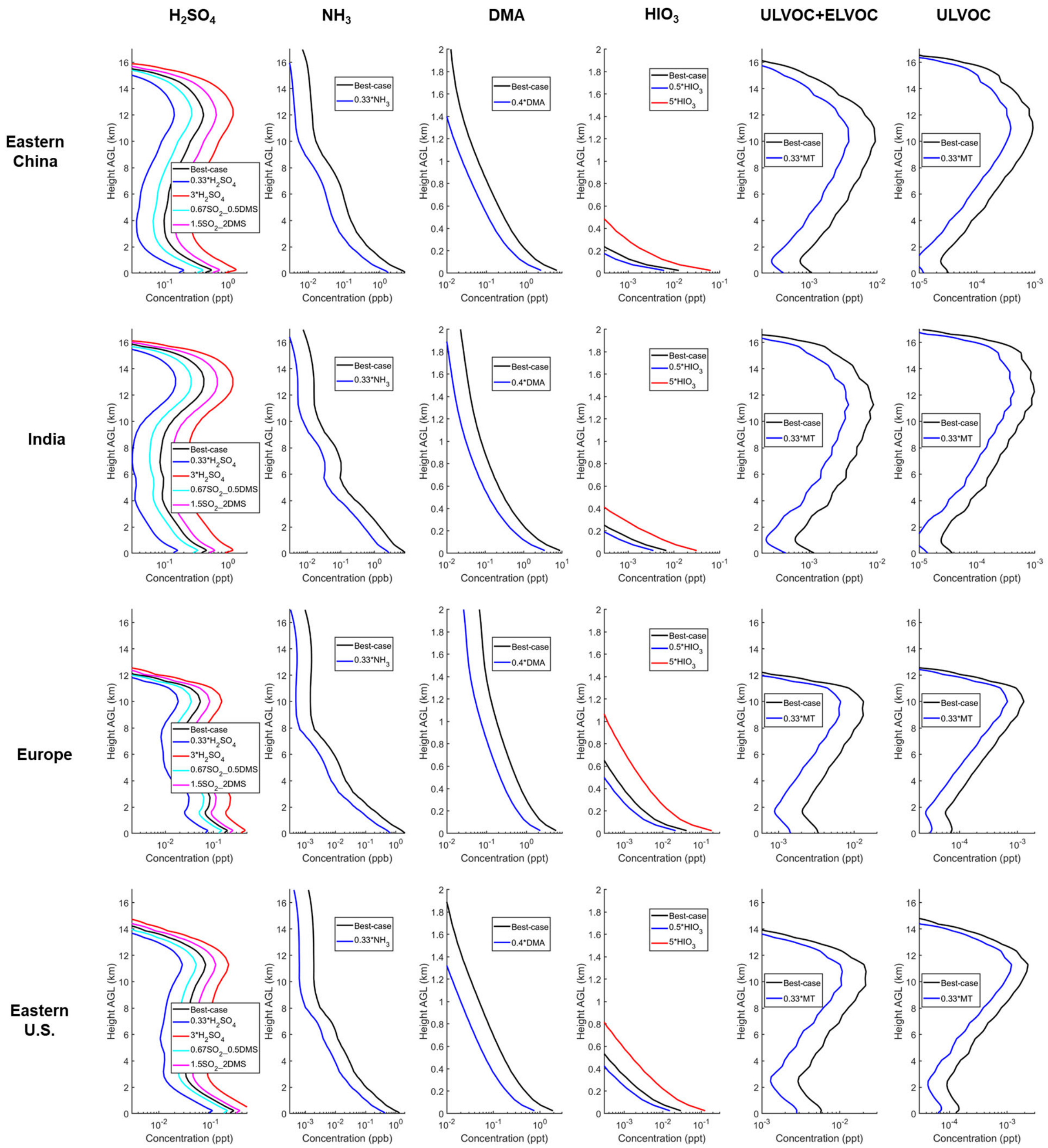

Supplementary Fig. 3 Same as Supplementary Fig. 2 but for anthropogenically polluted regions.

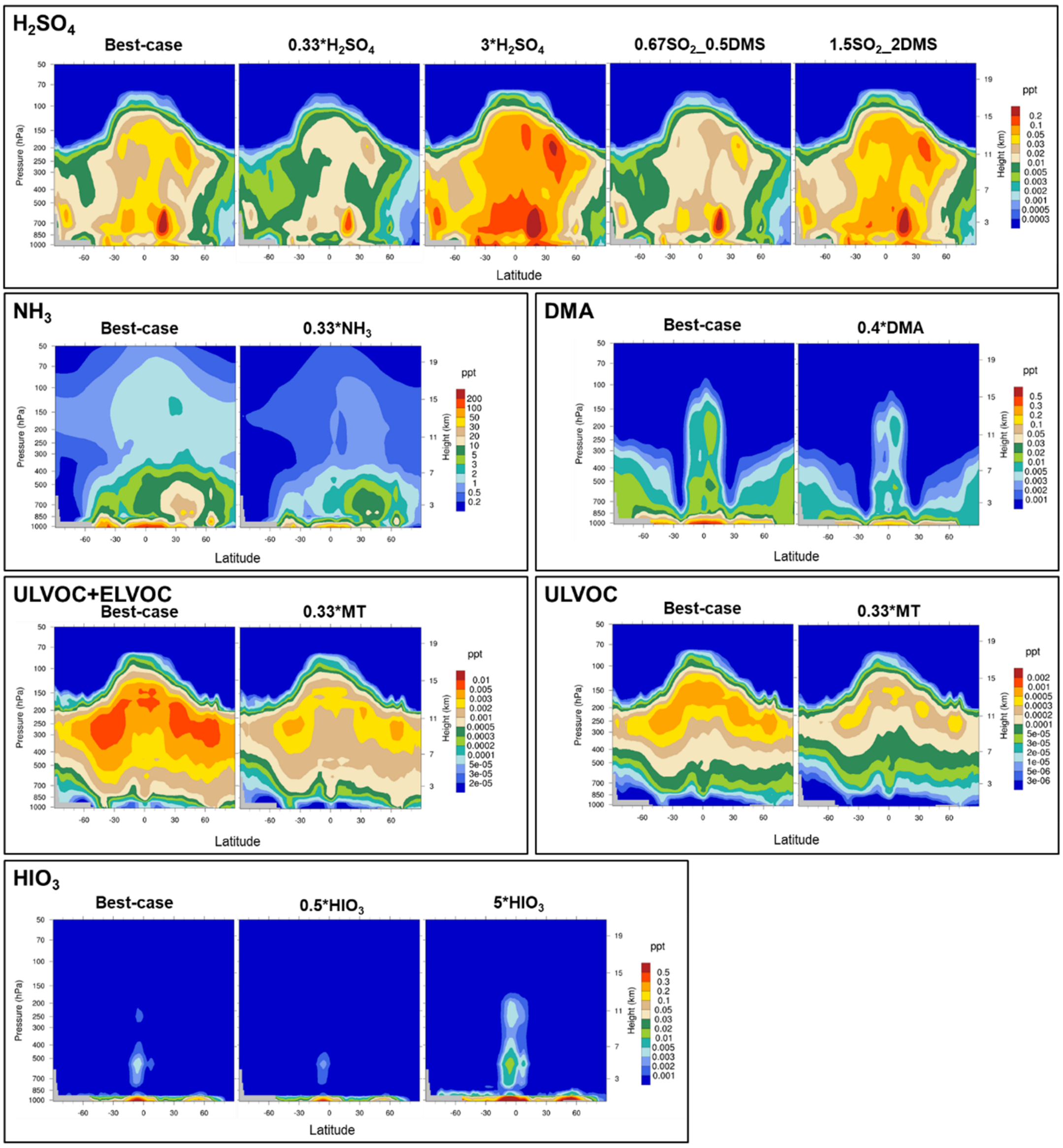

Supplementary Fig. 4 Zonal mean concentrations of precursors directly involved in nucleation over the Pacific Ocean (170°E–150°W) under the best-case and sensitivity scenarios in 2016. Definitions of the sensitivity experiments are presented in Methods and Supplementary Table 1. To make the figures concise, we show only the concentrations of a precursor in the best-case simulation and in the sensitivity simulations where its concentrations show significant differences from the best case.

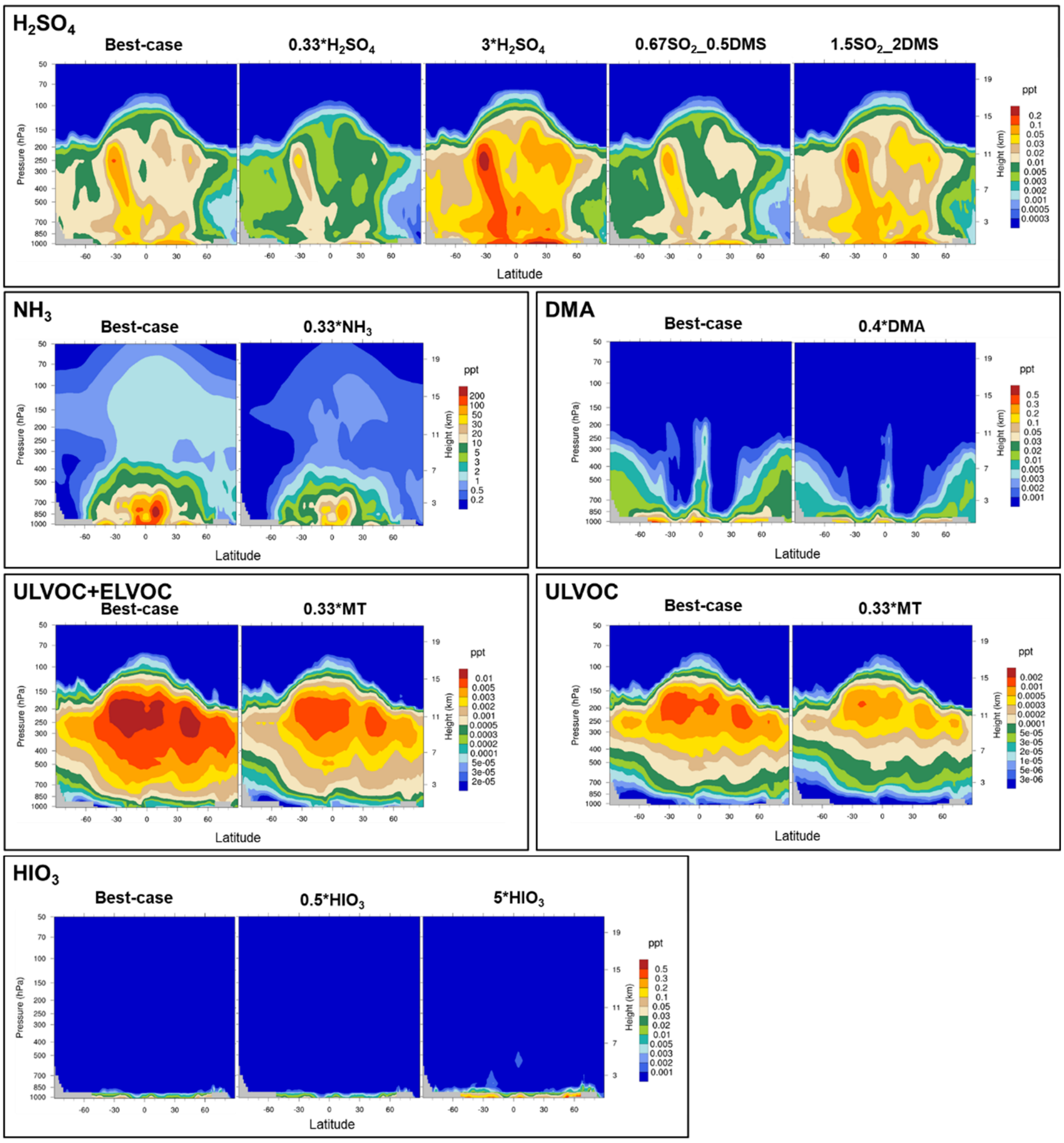

Supplementary Fig. 5 Same as Supplementary Fig. 4 but for the Atlantic Ocean (20°–40°W).

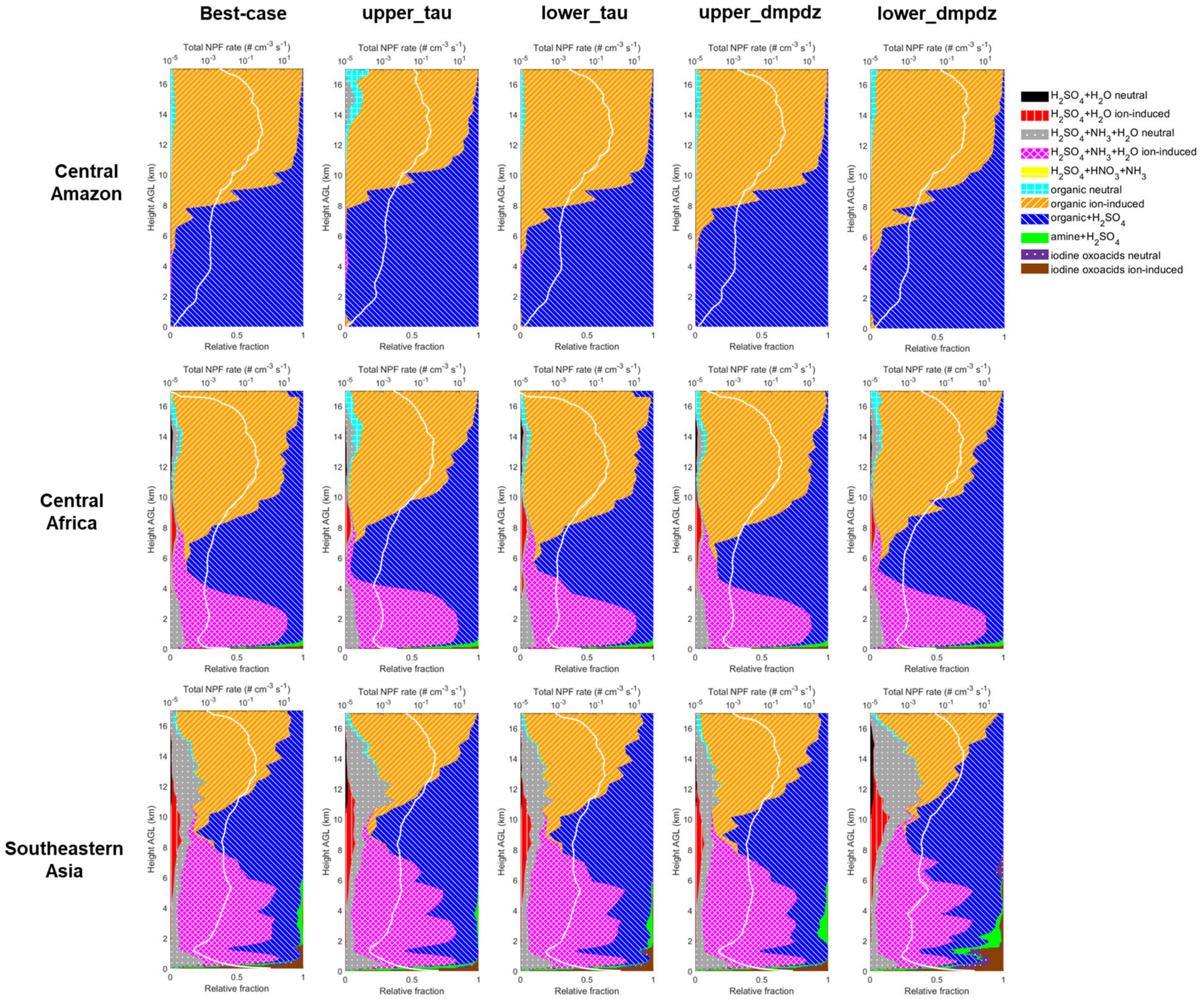

Supplementary Fig. 6 NPF rates as a function of height above ground level (AGL) over rainforests under the best-case scenario and sensitivity scenarios perturbing key parameters in the ZM deep convection scheme. White lines represent the total NPF rates of all mechanisms at diameter of 1.7 nm ($J_{1.7}$, on a log scale), and the colored areas represent the relative contributions of different mechanisms, both averaged in 2016 over the regions specified in Extended Data Fig. 1B. Definitions of the sensitivity experiments are presented in Methods and Supplementary Table 1.

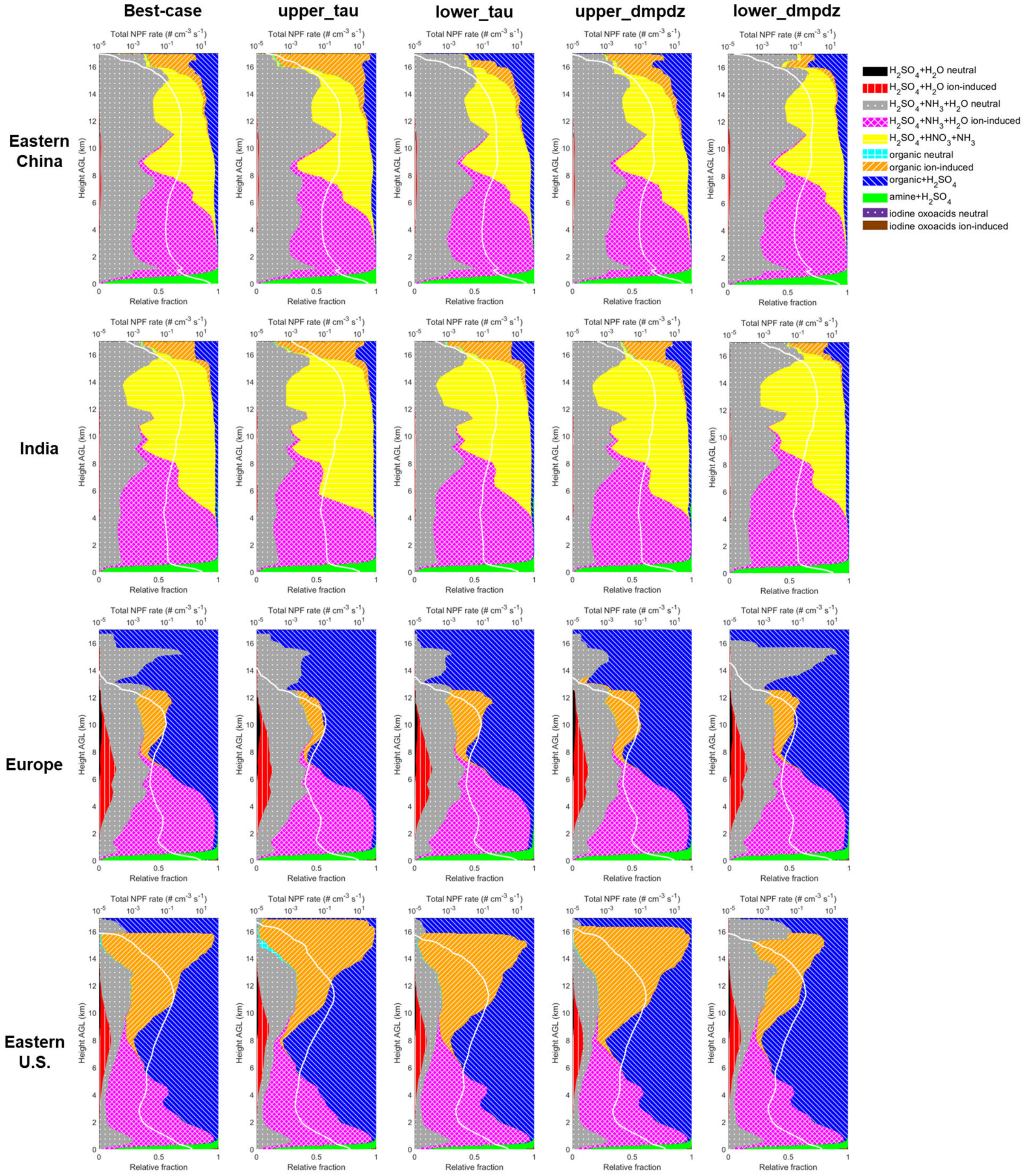

Supplementary Fig. 7 Same as Supplementary Fig. 6 but for anthropogenically polluted regions.

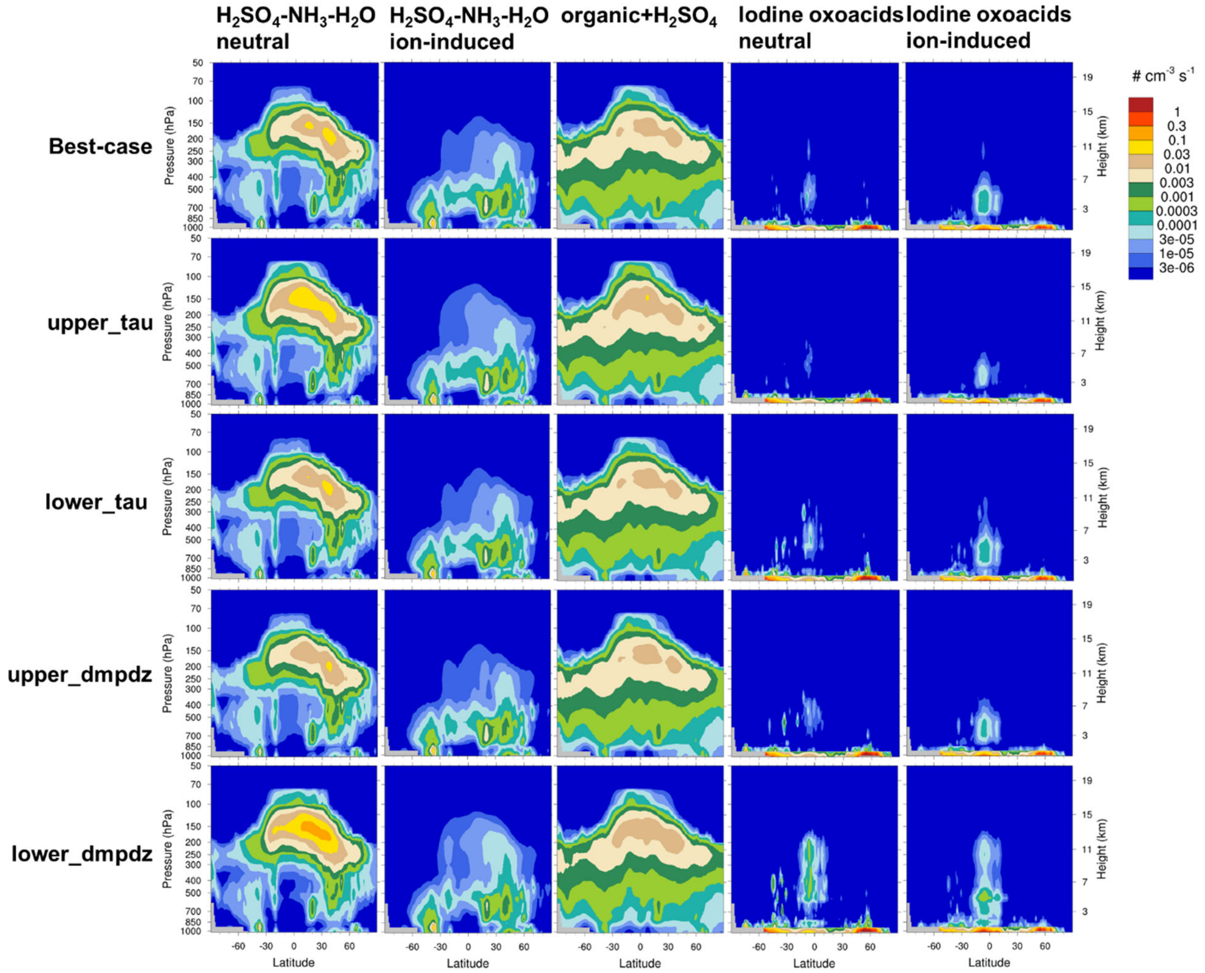

Supplementary Fig. 8 Zonal mean NPF rates of individual mechanisms over the Pacific Ocean (170°E–150°W) under the best-case scenario and sensitivity scenarios perturbing key parameters in the ZM deep convection scheme in 2016. Only five NPF mechanisms are shown because the other mechanisms are negligible in these regions. Definitions of the sensitivity experiments are presented in Methods and Supplementary Table 1.

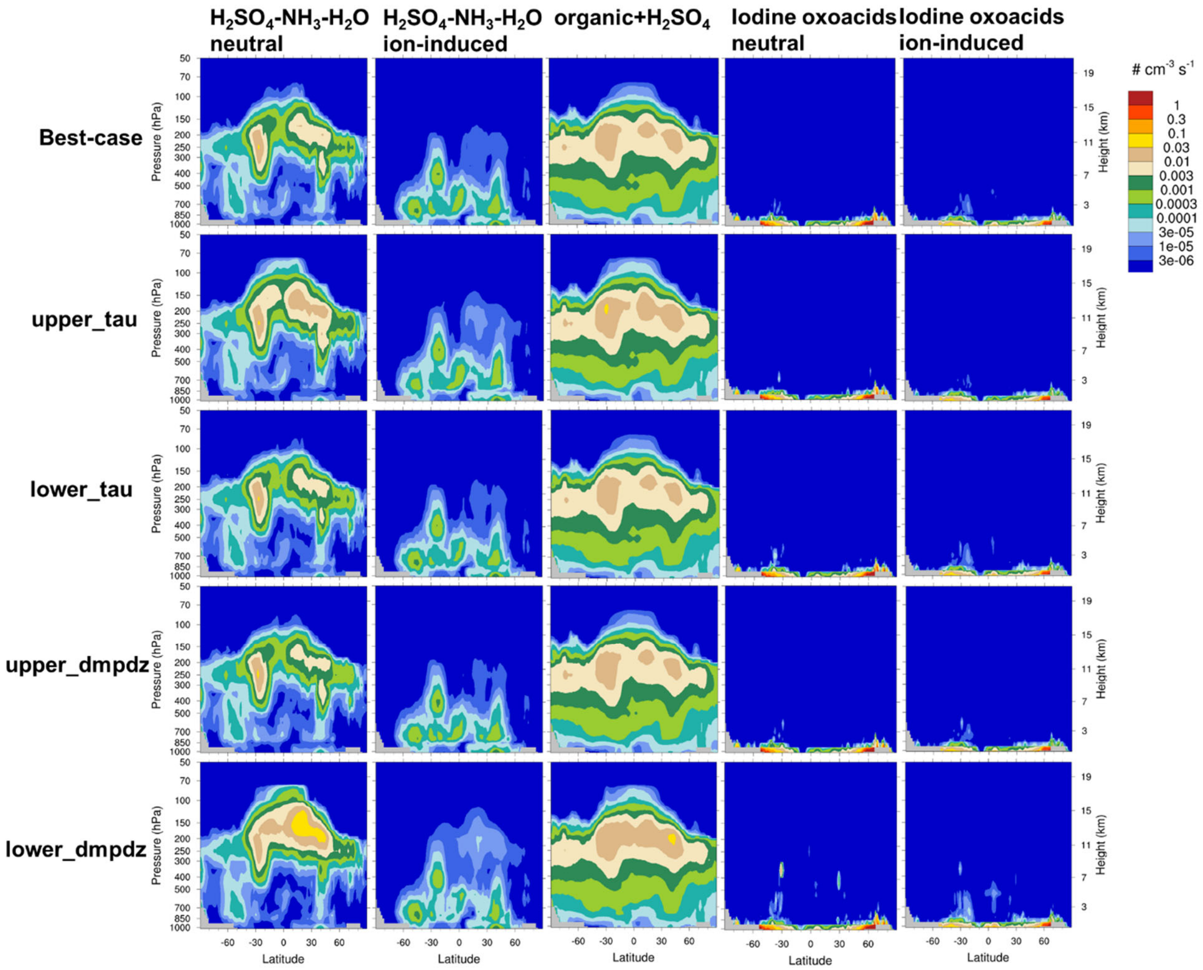

Supplementary Fig. 9 Same as Supplementary Fig. 8 but for Atlantic Ocean (20°–40°W).

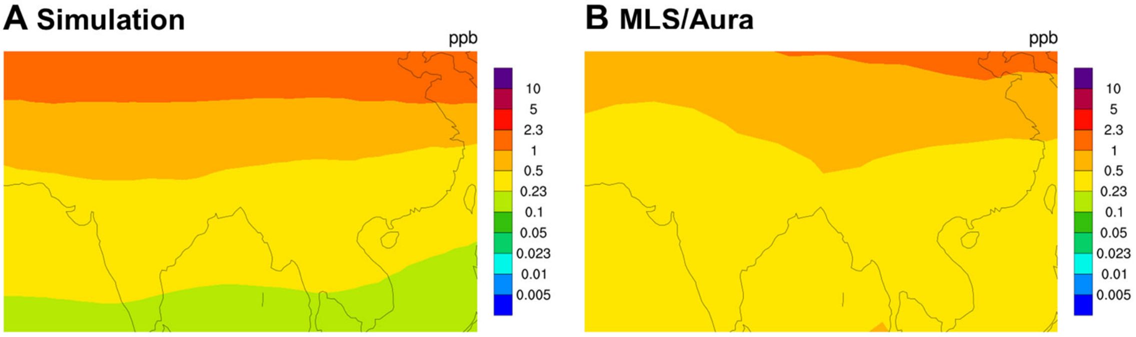

Supplementary Fig. 10 Comparison of simulated 2016 mean HNO_3 concentrations in the upper troposphere (150 hPa, approximately 13 km) over the Asian monsoon region with observations from the Microwave Limb Sounder (MLS) aboard the Aura satellite.

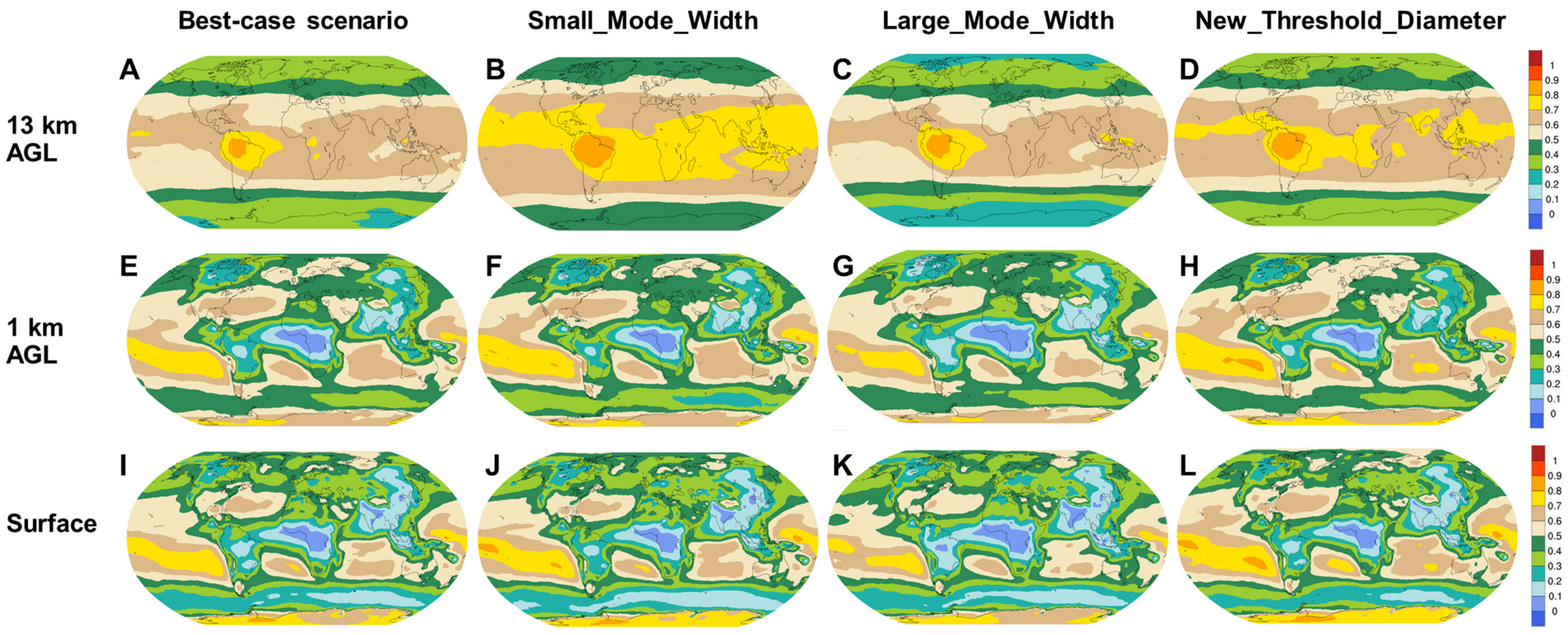

Supplementary Fig. 11 Fractions of CCN at 0.5% supersaturation (CCN0.5%) caused by NPF at different vertical levels in 2016 under different sensitivity simulations. (A–D) 13 km AGL, (E–H) 1 km AGL (approximately at the low-cloud level), and (I–L) surface level. The sensitivity experiments are defined in the section of "Configuration of the updated E3SM" in Methods.

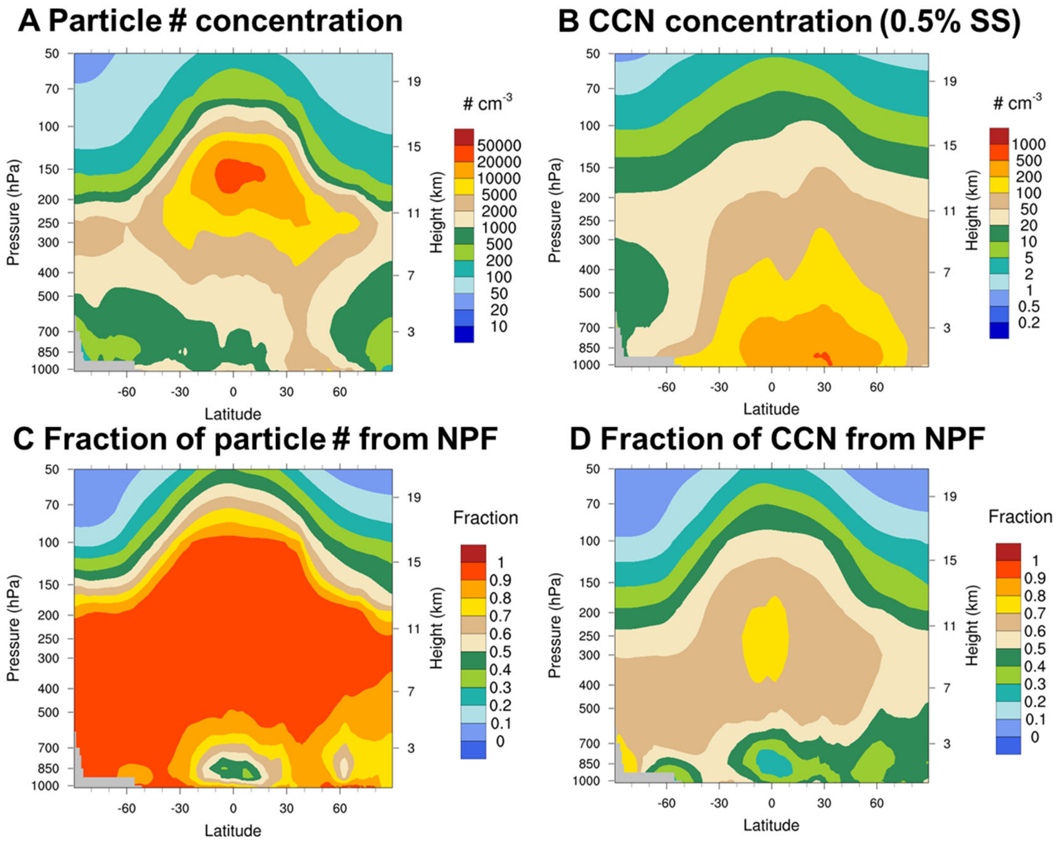

Supplementary Fig. 12 Zonal mean particle number and CCN concentrations and the fractions caused by NPF worldwide in 2016. (A, B) Zonal mean concentrations of **(A)** particle number of all sizes and **(B)** CCN0.5% from the best-case simulation. **(C, D)** Fractions of zonal mean concentrations of **(C)** particle number and **(D)** CCN0.5% caused by NPF. Particle number concentrations cover the entire size range (cf. field observations are mostly made for particles larger than a certain cutoff size). All concentrations are normalized to STP.

Supplementary Table 1 Summary of model scenarios developed in this study.

Scenario	Description
Best-case	A simulation that includes all 11 NPF mechanisms and uses the R2D-VBS to simulate the nucleating organics. This is our comprehensive best-case scenario and used in most analyses in this study.
No_NPF	A simulation that does not consider any NPF.
NPF_Mech4	A simulation that considers only four traditional inorganic nucleation mechanisms, i.e., the neutral and ion-induced H_2SO_4-H_2O mechanisms and H_2SO_4-NH_3-H_2O mechanisms, which resembles the NPF treatment in commonly used climate models.
NPF_Mech11_constYield	A simulation that includes all 11 NPF mechanisms but assumes that pure-organic and organic-H_2SO_4 nucleation is driven by a fixed fraction of the monoterpene oxidation products, following the treatment of a number of previous modeling studies[1-4]. The specific "fixed fractions" used here followed Gordon et al.[5]. Specifically, organic–H_2SO_4 nucleation was linked to all oxidation products of monoterpenes; in other words, the "fixed fraction" of monoterpene oxidation products used to drive organic–H_2SO_4 nucleation was 1.0. Pure-organic nucleation was assumed driven by highly oxygenated organic molecules (HOMs), the molar yields (fixed fraction) of which were assumed to be 1.4% for the reaction of monoterpenes with O_3 and 0.6% for the reaction of monoterpenes with OH.
0.67*SO_2_0.5*DMS	The same as "Best-case" except that the SO_2 and DMS emissions are reduced by a factor of 1.5 and 2, respectively.
1.5*SO_2_2*DMS	The same as "Best-case" except that the SO_2 and DMS emissions are increased by a factor of 1.5 and 2, respectively.
0.33*H_2SO_4	The same as "Best-case" except that the simulated H_2SO_4 concentrations are reduced by a factor of 3.
3*H_2SO_4	The same as "Best-case" except that the simulated H_2SO_4 concentrations are increased by a factor of 3.
0.33*MT	The same as "Best-case" except that the monoterpene emissions are reduced by a factor of 3.
org-weak-T-dependence	The same as "Best-case" except that a weaker temperature dependence of pure-organic and organic-H_2SO_4 nucleation rates is used.

organic-H_2SO_4_Riccobono	The same as "Best-case" except that the organic-H_2SO_4 nucleation parameterization is replaced with the one reported in Riccobono et al.[6].
0.4*DMA	The same as "Best-case" except that the simulated DMA concentrations are set to 0.4 times the original simulation results.
amine-H_2SO_4_Almeida	The same as "Best-case" except that the amine+H_2SO_4 nucleation rate parameterization directly derived from CLOUD chamber experiments reported by Almeida et al.[7] is used.
0.33*NH_3	The same as "Best-case" except that the NH_3 concentrations are reduced by a factor of 3.
nonuniform-NH_3	The same as "Best-case" except that the NH_3 concentration is 1 ppb (consistent with observations in the convective outflow hotspots by Höpfner et al.[8]) in [average NH_3]/1 ppb of the area of each model grid and zero in the remaining area of the model grid in the upper troposphere. For the areas with the presence of NH_3, we assume that H_2SO_4 is exhausted by nucleation.
0.5*HIO_3	The same as "Best-case" except that the simulated HIO_3 concentrations are reduced by a factor of 2.
5*HIO_3	The same as "Best-case" except that the simulated HIO_3 concentrations are increased by a factor of 5.
upper_tau	The time scale for the consumption rate of convective available potential energy (denoted by "tau") is set to the upper bound (14,400 s) of the possible range specified by Qian et al.[9], as compared to the value of 3,600 s in the best-case simulation.
lower_tau	The time scale for the consumption rate of convective available potential energy (denoted by "tau") is set to the lower bound (1,800 s) of the possible range specified by Qian et al.[9], as compared to the value of 3,600 s in the best-case simulation.
upper_dmpdz	The fractional mass entrainment rate (denoted by "dmpdz") is set to the upper bound (2.0×10^{-3} m^{-1}) of the possible range specified by Qian et al.[9], as compared to 0.7×10^{-3} m^{-1} in the best-case simulation.
lower_dmpdz	The fractional mass entrainment rate (denoted by "dmpdz") is set to the lower bound (0.1×10^{-3} m^{-1}) of the possible range specified by Qian et al.[9], as compared to 0.7×10^{-3} m^{-1} in the best-case simulation.

NPF_Mech4_scaled	In this scenario, we applied a fixed scaling factor to the NPF rates in NPF_Mech4 (which only includes four traditional nucleation mechanisms involving H_2SO_4, NH_3, and H_2O) such that its globally averaged NPF rate matched that of the best-case simulation. Here, the globally averaged NPF rate is defined as the average of the NPF rates across all model grid boxes (both horizontally and vertically), weighted by the volumes of those grid boxes.

Supplementary Table 2 Iodine chemical reactions implemented in the model.

**Gas−phase reactions**	
**Reaction**	**k (molec^{-1} cm^3 s^{-1})**
$I_2 + O \rightarrow IO + I$	$1.30 \cdot 10^{-10}$ [a]
$I + O_3 \rightarrow IO + O_2$	$2.00 \cdot 10^{-11} \cdot \exp(-830\ K/T)$
$IO + O \rightarrow I + O_2$	$1.40 \cdot 10^{-10}$ [a]
$IO + O_3 \rightarrow OIO + O_2$	$3.60 \cdot 10^{-16}$
$IO + IO \rightarrow OIO + I$	$2.13 \cdot 10^{-11} \cdot \exp(180\ K/T) \cdot$
	$(1+\exp(-p/19142\ Pa))$ [b]
$IO + IO \rightarrow IOIO$	$3.27 \cdot 10^{-11} \cdot \exp(180\ K/T) \cdot$
	$(1-0.65 \cdot \exp(-p/19142\ Pa))$ [b]
$IO + OIO \rightarrow I_2O_3$	$1.55 \cdot 10^{-10}$
$OIO + OIO \rightarrow I_2O_4$	$9.35 \cdot 10^{-11}$
$I_2 + OH \rightarrow HOI + I$	$1.80 \cdot 10^{-10}$
$HOI + OH \rightarrow IO + H_2O$	$2.00 \cdot 10^{-13}$
$IO + OH \rightarrow HO_2 + I$	$1.00 \cdot 10^{-10}$
$IO + HO_2 \rightarrow HOI$	$1.30 \cdot 10^{-11} \cdot \exp(570\ K/T)$
$I + HO_2 \rightarrow HI + O_2$	$1.50 \cdot 10^{-11} \cdot \exp(-1090\ K/T)$
$HI + OH \rightarrow I + H_2O$	$3.00 \cdot 10^{-11}$
$I_2 + O_3 \rightarrow IO + OIO$	$2.00 \cdot 10^{-15} \cdot \exp(-2050\ K/T)$
$I_2 + O_3 \rightarrow IO + I + O_2$	$2.00 \cdot 10^{-15} \cdot \exp(-2050\ K/T)$
$IOIO + O_3 \rightarrow IOIO_4$	$8.20 \cdot 10^{-15} \cdot \exp(763\ K/T)$
$IOIO_4 + H_2O \rightarrow HIO_3 + O_2 + HOI$	$2.50 \cdot 10^{-12} \cdot \exp(-2481\ K/T)$
$OIO + OH \rightarrow HIO_3$	$2.20 \cdot 10^{-10} \cdot \exp(243\ K/T)$
$I + NO_3 \rightarrow IO + NO_2$	$4.50 \cdot 10^{-10}$
$I_2 + NO_3 \rightarrow I + IONO_2$	$1.50 \cdot 10^{-12}$
$IONO_2 + I \rightarrow I_2 + NO_3$	$1.00 \cdot 10^{-10}$
$I + NO \rightarrow INO$	k_f [cd]
$I + NO_2 \rightarrow INO_2$	k_f [ce]
$IO + NO \rightarrow I + NO_2$	$8.60 \cdot 10^{-12} \cdot \exp(230\ K/T)$
$IO + NO_2 \rightarrow IONO_2$	k_f [cf]
$INO + INO \rightarrow I_2 + NO + NO$	$8.40 \cdot 10^{-11} \cdot \exp(-2620\ K/T)$
$INO_2 + INO_2 \rightarrow I_2 + NO_2 + NO_2$	$2.90 \cdot 10^{-11} \cdot \exp(-2600\ K/T)$
**Thermal decomposition**	

Reaction	k (s^{-1})
IOIO → OIO + I	$8.40 \cdot 10^{13} \cdot \exp(-12026\ K/T)$
IOIO → IO + IO	[bg]
I_2O_3 → OIO + IO	[h]
I_2O_4 → OIO + OIO	[bi]
$IONO_2$ → IO + NO_2	$1.10 \cdot 10^{15} \cdot \exp(-12060\ K/T)$
**Photolysis reactions**	
**Reaction**	j [j]
I_2 → I + I	$1.33 \cdot 10^{1} \cdot j(NO_2)$
IO → I + $O(^3P)$	$1.94 \cdot 10^{1} \cdot j(NO_2)$
OIO → I + O_2	$4.59 \cdot 10^{1} \cdot j(NO_2)$
IOIO → IO + IO	$6.22 \cdot j(NO_2)$
I_2O_3 → OIO + IO	$2.14 \cdot j(NO_2)$
I_2O_4 → OIO + OIO	$7.45 \cdot j(NO_2)$
HOI → I + OH	$9.18 \cdot 10^{-1} \cdot j(NO_2)$
HI(+O_2) → HO_2 + I	$1.12 \cdot 10^{-3} \cdot j(NO_2)$
INO → I + NO	$3.27 \cdot j(NO_2)$
INO_2 → I + NO_2	$3.06 \cdot 10^{-1} \cdot j(NO_2)$
$IONO_2$ → I + NO_3	$4.90 \cdot j(NO_2)$

[a] The reaction is predicted to be negligible at the altitudes of interest

[b] p in the equation is fixed at 101,325 Pa because of its minor effect

[c] k_f is effective second-order rate constant defined at http://jpldataeval.jpl.nasa.gov

[d] $k_0 = 1.8 \cdot 10^{-32}$, $n = 1.0$, $k_\infty = 1.7 \cdot 10^{-11}$, $m = 0$, $[M]$ = [air]

[e] $k_0 = 3.0 \cdot 10^{-31}$, $n = 1.0$, $k_\infty = 6.6 \cdot 10^{-11}$, $m = 0$, $[M]$ = [air]

[f] $k_0 = 7.7 \cdot 10^{-31}$, $n = 3.5$, $k_\infty = 7.7 \cdot 10^{-12}$, $m = 1.5$, $[M]$ = [air]

[g] $k = (2.55335 \cdot 10^{11} - 4.41888 \cdot 10^{7} \cdot 0.75p/\mathrm{Pa} + 8561.86 \cdot (0.75 \cdot p/\mathrm{Pa})^2 + 1.421881 \cdot 10^{-2} \cdot (0.75p/\mathrm{Pa})^3) \cdot \exp((-11466.82304 + 597.01334 \cdot \exp(-0.75 \cdot p/\mathrm{Pa}/138262.325) - 167.3391 \cdot \exp(-0.75 \cdot p/\mathrm{Pa}/4375.089))\ K/T)$

[h] I_2O_3 is predicted to be thermally stable

[i] $k = (-1.92626 \cdot 10^{14} + 4.67414 \cdot 10^{11} \cdot 0.75p/\mathrm{Pa} - 36865.1 \cdot (0.75 \cdot p/\mathrm{Pa})^2 - 3.09109 \cdot (0.75p/\mathrm{Pa})^3) \cdot \exp((-12302.15294 + 152.78367 \cdot \exp(-0.75p/\mathrm{Pa}/4612.733) + 437.62868 \cdot \exp(-0.75 \cdot p/\mathrm{Pa}/42844.13))\ K/T)$

[j] $j(NO_2)$ is the photolysis rate of NO_2 (NO_2 → NO + $O(^3P)$)

Supplementary Table 3 Henry's law constants and aerosol uptake coefficients for iodine species implemented in the model.

Species	Henry's law constant (H) at 298K (mol m^{-3} atm^{-1})	d(ln$H$)/ d(1/T) (K)	Reference	Aerosol uptake coefficient	Reference
I_2	2.80×10^{3}	3900	The values are obtained from Karagodin-Doyennel et al.[10], except that the values for $IOIO_4$ and HIO_3 are assumed to be the same as those of I_2O_4 and HOI due to the lack of experimental data.	–	–
HOI	4.10×10^{5}	0		0.23	Li et al.[11], geometric mean of largest and smallest values used in the simulations ([0.06, 0.9]).
HI	7.30×10^{16}	3190		0.1	Sherwen et al.[12]
I	7.90×10^{1}	0		–	–
IO	∞	0		–	–
OIO	∞	0		–	–
IOIO	∞	0		0.02	Badia et al.[13]
I_2O_3	∞	0		0.02	Badia et al.[13]
I_2O_4	∞	0		0.02	Badia et al.[13]
$IOIO_4$	∞	0		0.02	Assumed to the same as I_2O_4
HIO_3	4.10×10^{5}	0		0.2	Estimated based on Finkenzeller et al.[14]
INO	3.00×10^{2}	0		–	–
INO_2	3.00×10^{2}	0		0.02	Badia et al.[13]
$IONO_2$	∞	0		0.01	Badia et al.[13]

Supplementary References:

1 Gordon, H. *et al.* Causes and importance of new particle formation in the present-day and preindustrial atmospheres. *J. Geophys. Res-Atmos.* **122**, 8739-8760, doi:10.1002/2017jd026844 (2017).

2 Scott, C. E. *et al.* The direct and indirect radiative effects of biogenic secondary organic aerosol. *Atmos. Chem. Phys.* **14**, 447-470, doi:10.5194/acp-14-447-2014 (2014).

3 Yu, F., Luo, G., Nadykto, A. B. & Herb, J. Impact of temperature dependence on the possible contribution of organics to new particle formation in the atmosphere. *Atmos. Chem. Phys.* **17**, 4997-5005, doi:10.5194/acp-17-4997-2017 (2017).

4 Reddington, C. L. *et al.* Primary versus secondary contributions to particle number concentrations in the European boundary layer. *Atmos. Chem. Phys.* **11**, 12007-12036, doi:10.5194/acp-11-12007-2011 (2011).

5 *Beijing Air Quality Bulletin*, <http://www.bjee.org.cn/cn/index.php> (

6 Riccobono, F. *et al.* Oxidation Products of Biogenic Emissions Contribute to Nucleation of Atmospheric Particles. *Science* **344**, 717-721, doi:10.1126/science.1243527 (2014).

7 Almeida, J. *et al.* Molecular understanding of sulphuric acid-amine particle nucleation in the atmosphere. *Nature* **502**, 359-363, doi:10.1038/nature12663 (2013).

8 Hoepfner, M. *et al.* Ammonium nitrate particles formed in upper troposphere from ground ammonia sources during Asian monsoons. *Nat. Geosci.* **12**, 608-612, doi:10.1038/s41561-019-0385-8 (2019).

9 Qian, Y. *et al.* Parametric Sensitivity and Uncertainty Quantification in the Version 1 of E3SM Atmosphere Model Based on Short Perturbed Parameter Ensemble Simulations. *J. Geophys. Res-Atmos.* **123**, 13046-13073, doi:10.1029/2018jd028927 (2018).

10 Karagodin-Doyennel, A. *et al.* Iodine chemistry in the chemistry-climate model SOCOL-AERv2-I. *Geosci. Model. Dev.* **14**, 6623-6645, doi:10.5194/gmd-14-6623-2021 (2021).

11 Li, Q. *et al.* Role of Iodine Recycling on Sea-Salt Aerosols in the Global Marine Boundary Layer. *Geophys. Res. Lett.* **49**, e2021GL097567, doi:10.1029/2021gl097567 (2022).

12 Sherwen, T. *et al.* Iodine's impact on tropospheric oxidants: a global model study in GEOS-Chem. *Atmos. Chem. Phys.* **16**, 1161-1186, doi:10.5194/acp-16-1161-2016 (2016).

13 Badia, A. *et al.* Importance of reactive halogens in the tropical marine atmosphere: a regional modelling study using WRF-Chem. *Atmos. Chem. Phys.* **19**, 3161-3189, doi:10.5194/acp-19-3161-2019 (2019).

14 Finkenzeller, H. *et al.* The gas-phase formation mechanism of iodic acid as an atmospheric aerosol source. *Nat. Chem.* **15**, 129-135, doi:10.1038/s41557-022-01067-z (2022).
